# A novel polysaccharide of *Citrus medica* L. var. *sarcodactylis* Swingle: purification, structural characterization, and hypolipidemic effects

**DOI:** 10.1016/j.fochx.2026.103559

**Published:** 2026-01-17

**Authors:** Chong Wu, Lianger Dong, Jinhua Zhou, Sibao Wan, Zhen Qin, Haiyan Gao

**Affiliations:** a School of Environmental and Chemical Engineering, Shanghai University, Shanghai, China; b School of Life Sciences, Shanghai University, Shanghai 200444, China; c Shanghai Key Laboratory of Bio-Energy Crops, School of Life Sciences, Shanghai University, Shanghai 200444, China

**Keywords:** Fingered citron, Polysaccharide, Structural characterization, Hypolipidemic activity

## Abstract

Fingered citron (*Citrus medica* L. var. *sarcodactylis* Swingle) is an edible fruit with great potential in functional food. In this study, we isolated and purified a novel polysaccharide from fingered citron, designated as ZFP2, and investigated its structural characteristics and in vitro hypolipidemic activity. ZFP2 was found to be composed of arabinose (Ara), galactose (Gal), and galacturonic acid (GalA) in a molar ratio of 2.61:2.27:14.39, with an average molecular weight of 407 kDa. The majorly backbone of ZFP2 is composed of 1,4-GalpA, 1,2,4-Rhap, 1,5-Araf, 1,6-Galp and 1,3,6-Galp. ZFP2 exhibited a semi-crystalline structure and irregular plate-like morphology. In an oleic acid (OA)-induced lipid overload model using human liver cancer cells (HepG2), ZFP2 significantly reduced intracellular lipid accumulation and improved lipid profiles. Mechanistic studies demonstrated that ZFP2 modulates lipid metabolism through the *PPARα/LXRα/ABCG8* signaling pathway, enhancing lipid catabolism and reducing lipid accumulation. These findings underscore the potential of ZFP2 as a natural hypolipidemic agent and support the development of fingered citron polysaccharide as functional food ingredients for managing lipid-related disorders.

## Highlights

• A novel polysaccharide (ZFP2) was isolated from *Citrus medica* L. var. *sarcodactylis* Swingle.
• The structure of ZFP2 was characterized.
• ZFP2 alleviated cellular lipid accumulation and mitigated oxidative stress.
• ZFP2 modulated the expression of key genes within pathways associated with lipid metabolism.

## 1 Introduction

Hyperlipidemia is primarily manifested by elevated plasma total cholesterol (TC), triglycerides (TG), high-density lipoprotein cholesterol (HDLC) and low-density lipoprotein cholesterol (LDLC) levels. It is a significant risk factor for metabolic syndromes and cardiovascular diseases. Each year, approximately 17 million people worldwide lose their lives to cardiovascular diseases, and hyperlipidemia accounts for 30% of the fatalities (Sonnenburg & Bäckhed, 2016). Therefore, timely prevention and treatment of hyperlipidemia are crucial. While pharmaceutical treatments are available, they often come with side effects such as muscle and liver damage. As a result, there has been an increasing interest in natural alternatives such as plant-derived polysaccharides, which have demonstrated potential in preventing and managing hyperlipidemia (Ji et al., 2020; Kalita et al., 2022).

Fingered citron (*Citrus medica* L. var*. sarcodactylis* Swingle), commonly known as Buddha's hand, is an evergreen shrub or small tree of the citrus family *Rutaceae*. Native to northwestern India, it has been widely used in East Asian cuisine. In addition to being processed into desserts, drinks, candies, and sauces, fingered citron has been used traditionally to treat ailments such as stomachache, infectious diseases, hypertension, and arthritis among Asia countries like Japan and China (Sun et al., 2024). In recent years, the chemical constituents isolated from the fruits of fingered citron include polysaccharides, neolignans, flavonoids, coumarins, terpenoids, glycosides, and other bioactive substances (Ghani et al., 2022; Wang et al., 2020), which have revealed a wide variety of biological activities, including hypolipidemic, immunoregulatory (Luo et al., 2022), hepatoprotective, antibacterial, anticancer, antioxidant, antihyperglycemic (Yang et al., 2022), gastroprotective effect, antidepressant and cardioprotective activities.

Polysaccharides are among the primary active components of fingered citron. Fingered citron polysaccharides (FCPs) have garnered a significant interest from researchers due to their potential health benefits and notable bioactivities. FCPs could be extracted from diverse parts of the fingered citron plant, including its peels, fruits and leaves. Typically, the raw materials utilized for FCPs extraction consist of the flesh or the peel of the fresh or the dried fingered citron fruits, as well as the processed fruit residues. The current challenges (Sun et al., 2024) mainly include: selecting an appropriate extraction method to effectively separate FCPs while maintaining its structural integrity; clarifying the structural characteristics of FCPs through complex analytical methods, including monosaccharide composition, glycosidic bonds, branching patterns, and molecular weight distribution; accurately evaluating the biological activity of FCPs and elucidating the underlying mechanism of action. Research should reveal the unique pharmacological mechanisms of FCPs and pave the way for advanced therapeutic interventions and functional food formulations. Previous studies have explored their extraction methods, structural features (Shuhui et al., 2023), antioxidant properties, immune-boosting activities (Bao et al., 2019). The impact of FCPs on glycolipid metabolism should not be overlooked. Xia et al. discovered that FCPs exhibited a specific affinity for sodium cholate, sodium taurocholate, and sodium glycocholate, thereby confirming their lipid-lowering effects (Sun et al., 2024). Yang et al. identified that FCP-2-1 could exert its hypoglycemic activity by inhibiting the activity of related enzymes and improving the metabolism of sugar and lipid (Yang et al., 2022). However, research on FCPs has mainly focused on crude polysaccharides, with a lack of evidence clarifying their specific structures. Additionally, there remains a dearth of research on the specific mechanisms through which citrus polysaccharides and fingered citron polysaccharides exert their hypolipidemic effects.

Previous studies have already demonstrated that polysaccharides derived from citrus fruits exhibit hypolipidemic effects, presumably attributed to their ability to enhance cholesterol and bile acid excretion (Chau et al., 2004). Zeng et al. demonstrated that the polysaccharide FMPS from *Fortunella margarita* (Lour.) Swingle possesses in vitro inhibitory activity against pancreatic lipase, bile acid-binding capacity, and antioxidant activity, indicating its potential for lipid-lowering effects. And the hypolipidemic effect of FMPS in hyperlipidemic rats is achieved by reducing lipid content and enhancing the activity of antioxidant enzymes (Zeng et al., 2019). Méndez-Albiñana et al. (Méndez-Albiñana et al., 2025) identified a pectic polysaccharide with a molecular weight of 429 kDa from a Spanish citrus variety, which is mainly composed of xylose (Xyl), arabinose (Ara), galactose (Gal), and galacturonic acid (GalA). In vivo experiments confirmed that this polysaccharide can reduce weight gain and decrease serum total cholesterol (TC) and low-density lipoprotein cholesterol (LDLC) levels in rats fed a high-fat diet. The above studies collectively indicate that citrus polysaccharides have significant lipid-lowering potential. However, further research on their specific hypolipidemic mechanisms remains insufficient.

In this study, a novel polysaccharide was extracted and purified from fingered citron, and its structure was clearly characterized. Cell experiments demonstrated that ZFP2 could alleviate hyperlipidemia by reducing oxidative stress and lipid accumulation. We further identified that this activity involved regulation of the PPARα/LXRα/ABCG8 signaling pathways, providing a mechanistic insight into its hypolipidemic effect.

## 2 Materials and methods

### 2.1 Plant materials and chemicals

Dried fingered citron fruits were purchased from Jinhua, Zhejiang province, China. Samples were ground into powder using a grinder, sieved through a 40-mesh sieve, and stored in −20 °C for further use. Human liver cancer cells were purchased from Hunan Fenghui Biotechnology Co., Ltd. (Hunan, China). Trypsin, fetal bovine serum (FBS) and Dulbecco's Modified Eagle's medium (DMEM) were purchased from Thermo Fisher Scientific (MA, USA). Assay kits for TG, TC, LDLC, HDLC, malondialdehyde (MDA), superoxide dismutase (SOD) and total antioxidant capacity (T-AOC) were obtained from Nanjing Jiancheng Bioengineering Institute (Nanjing, China). Bicinchoninic acid (BCA) protein concentration assay kit, radio immunoprecipitation assay (RIPA) cell lysis buffer, methylthiazolyldiphenyl-tetrazolium bromide (MTT) kit, first-strand cDNA synthesis mix kit, and goat anti-rabbit IgG were purchased from Shanghai Biyuntian Biotechnology Co., Ltd. (Shanghai, China). β-Actin rabbit monoclonal antibody, rabbit polyclonal antibody (PPARα, LXRα, SHP, ABCG8, FXR) were purchased from Wuhan Aibotaike Biotechnology Co., LTD. (Wuhan, China). Hieff qPCR SYBR Green Master Mix were purchased from Yisheng Biotechnology Co., LTD. (Shanghai, China). The Tanon™ electro chemiluminescence (ECL) substrate was purchased from Shanghai Tianneng Life Science Co., LTD. (Shanghai, China). Dextran standards (5.2 kDa, 11.6 kDa, 23.8 kDa, 48.6 kDa, 148 kDa, 273 kDa, 410 kDa, and 668 kDa) were purchased from Sigma-Aldrich (St. Louis, MO, USA). All chemicals used were of analytical grade.

### 2.2 Preparation of crude polysaccharide (ZFP)

The extraction method of ZFP was referred to a previous study (Peng, Yang, et al., 2019). These extraction conditions are adopted to maximize the retention of the original structure of the polysaccharide while achieving the highest possible yield. Dried fingered citron powder (100 g) was treated with anaqueous ethanol (1:10, *w*/*v*) at 70 °C for 1 h to remove alcohol-soluble substances. After filtering, the residue was added to ultrapure water at a liquid-to-material ratio of 1:20 (*w*/*v*), and the mixture was heated at 90 °C for 1.5 h. The filtrate was then concentrated at 60 °C. The concentrated solution was deproteinized with Sevage reagent (chloroform/n-butanol, 4:1, *v*/v). The resulting solution was mixed with anaqueous alcohol overnight, and then collected for the precipitate by centrifuging at 5777 ×*g* for 20 min. The resulting sediment was re-dissolved in water and then freeze-dried for at least 48 h to yield ZFP.

### 2.3 Isolation and purification of ZFP

The purification method of ZFP was referenced from previous studies with slight modifications (Ji et al., 2020; Kalita et al., 2022). ZFP was purified on a Q Ssepharose fast flow chromatographic column (2.6 cm × 40 cm), eluted with ultra-pure water, 0.1, 0.2 and 0.3 M sodium chloride solutions at a flow rate of 2 mL/min. The phenol‑sulfuric acid method was used to measure the polysaccharide content. Four fractions were then collected based on the elution profile and labeled as ZFP0, ZFP1, ZFP2 and ZFP3. The major fraction ZFP2 was further purified on a Sepharose CL-6B chromatography column (1.6 × 50 cm), eluted with ultra-pure water at a flow rate of 0.6 mL/min. ZFP2 powder was obtained by lyophilization. More detailed operating conditions for polysaccharide purification were shown in the supplementary materials.

### 2.4 Characterization of ZFP2

#### 2.4.1 Chemical analysis

Refer to previous studies (Pan et al., 2022), The neutral sugar content was analyzed with a d-glucose standard using the phenol‑sulfuric acid method. Uronic acid content was measured with a glucuronic acid standard using the *m*-hydroxybiphenyl method. Protein content was determined using a bovine serum albumin (BSA) standard and the Coomassie brilliant blue method.

#### 2.4.2 Molecular weight

The molecular weight of polysaccharide samples was determined by high-performance gel permeation chromatography (HPGPC) (Wang et al., 2024). The weight-average molecular weight (Mw) is a molecular weight result calculated with mass as the weighting factor, and the number-average molecular weight (Mn) is a molecular weight result calculated with the number of molecules as the weighting factor. The ratio of the weight-average molecular weight to the number-average molecular weight is the polydispersity index (Mw/Mn). Samples were prepared into 5 mg/mL solutions, and the supernatants were filtered through a 0.22 μm membrane filter before being transferred to injection vials. Chromatographic conditions were as follows: chromatographic column: BRT105–104-102 series gel columns (8 × 300 mm); mobile phase: 0.05 M NaCl solution; flow rate: 0.6 mL/min; column temperature: 40 °C; injection volume: 20 μL; detector: refractive index detector RI-10 A.

#### 2.4.3 Monosaccharide composition

Monosaccharide composition (Ji et al., 2020; Kalita et al., 2022) was analyzed using an Ion chromatography system (ICS-5000 HPAEC) equipped with a pulsed amperometric detector (Thermo Fisher Scientific Inc., USA). ZFP2 was hydrolyzed with trifluoroacetic acid (3.0 mol/L, 120 °C, 3 h) and concentrated by rotary evaporation. After filtration through a 0.22 μm millipore filter, the sample was injected into a Dionex™ CarboPac™ PA20 IC column (3 × 150 mm) for gradient elution, mobile phase A: NaOH solution (15 mM), mobile phase B: NaOH solution (15 mM) and sodium acetate solution (100 mM). The flow rate is 0.3 mL/min and the temperature is 30 °C.

#### 2.4.4 Fourier transform infrared (FT-IR) spectroscopy analysis

The FT-IR spectrum of ZFP2 was obtained using a VERTEX70 spectrophotometer (Bruker Scientific Instruments LTD, Hong Kong, China). The determination method refers to previous studies (Wang et al., 2024). ZFP2 was grinded with potassium bromide power and pressed into pellets for spectrometric measurement in the range of 4000–400 cm^−1^.

#### 2.4.5 Nuclear magnetic resonance (NMR) spectroscopy analysis

ZFP2 was dissolved in 1 mL of deuterium oxide (D_2_O), freeze-dried, and subjected to deuterium-exchange by dissolving and freeze-drying in D_2_O three times. The final sample was dissolved in 1 mL D_2_O and transferred into an NMR tube. The analysis was performed according to the reported method (Peng, Luo, et al., 2019). One-dimensional (^1^H and ^13^C) and two-dimensional (Yao et al., 2021) (^1^H-^1^HCosy and HSQC) NMR spectra were recorded using a Bruker NMR spectrometer (STA449 F5).

#### 2.4.6 Methylation and GC–MS analysis

After carboxyl reduction, methylation, acid hydrolysis and acetylation, the glycosidic linkages of ZFP2 were identified by GC–MS (Qiao et al., 2024). Briefly, ZFP2 sample (10 mg) were dissolved in pure water (1 mL), and reacted with carbodiimide for 2 h (1 mL, 100 mg/mL), respectively, and was followed by reacted with imidazole (1 mL, 2 mol/L), NaBH_4_ (30 mg/mL) and NaBD_4_ (30 mg/mL) for 3 h. The reaction was terminated by glacial acetic acid (100 μL), and the samples were dialyzed for 48 h and freeze-dried. Then, the samples were redissolved in DMSO (500 μL), reacting with NaOH (1 mg) for 30 min and iodomethane solution (50 μL) for 1 h. After that, water and dichloromethane were added, mixed, and centrifuged, which was repeated three times, and then the dichloromethane phase was collected and dried. The processed samples were dissolved with TFA (100 μL, 2 mol/L), and hydrolyzed at 121 °C for 90 min, respectively. After hydrolysis, the samples were dried at 30 °C, and then reacted with ammonia (50 μL, 2 mol/L) and NaBD_4_ (1 mol/L) at room temperature for 2.5 h. The reaction was terminated by acetic acid, whose samples were dried with nitrogen, washed twice with methanol and reacted with acetic anhydride (250 μL) at 100 °C for 2.5 h. The samples were finally mixed with dichloromethane, washed with pure water three times, and the dichloromethane phase was detected.

The Agilent 7890 A gas chromatographic system (Agilent Technologies, USA) with HP-5MS capillary column (30 m × 0.25 mm × 0.25 μm, Agilent J&W Scientific, Folsom, CA, USA) was adopted. The carrier gas was helium (purity ≥99.99%), with a flow rate of 1.0 mL/min, and the inlet temperature was 260 °C. The injection volume was set to 1 μL, as well as split injection was performed with a split ratio of 10:1 and solvent delay of 2.2 min. The temperature programming was set as follows: keeping 50 °C for 1 min, raising to 130 °C at a rate of 50 °C/min, then raising to 230 °C at a rate of 3 °C/min, maintaining for 2 min. The Agilent 5977B Quadrupole mass spectrometry (Agilent Technologies, USA) with electron bombardment ion source and Mass Hunter workstation was applied. The inlet and quaternary rod temperatures were 230 °C, and 150 °C, respectively. SCAN mode with a quality scanning range (*m*/*z*) of 30–600 was set to collect the mass spectrum signals.

#### 2.4.7 X-ray diffraction (XRD) analysis

The XRD analysis method refers to the previous literature (Li et al., 2022). The crystalline structure of ZFP2 was examined using a polycrystalline X-ray diffractometer (Dandong Haoyuan Instrument Co., LTD, Liaoning, China). The analysis was carried out with an initial scanning angle is 5° and a final angle of 55°, using a stepping interval of 0.03°. The scan was set at a sampling time of 0.3 min per step, with the operating voltage at 40 kV and current at 40 mA.

#### 2.4.8 Scanning electron microscopy (SEM) analysis

The SEM analysis method refers to the previous literature (Peng, Luo, et al., 2019). The sample was evenly spread on the conductive adhesive, and any excess sample was removed using an earwash ball. The ZFP2 samples were then placed in a vacuum ion sputtering unit for gold coating for 90 s. The morphological features were observed using a scanning electron microscope (Phenom G2 Pro, Netherlands).

### 2.5 Hypolipidemic activities

#### 2.5.1 Cell culture

Cell culture was conducted by referring to previous studies (Liu et al., 2023). The cells were cultured in DMEM supplemented with 10% FBS and 1% penicillin/streptomycin at 37 °C under a humidified atmosphere of 5% CO2 for 24 h in an incubator (HCP-168, Qingdao Haier Biological Medical Co., LTD, Qingdao, China).

#### 2.5.2 Cell viability assay and induction of hepatic lipid overload

Refer to previous studies (Jiao et al., 2020), after incubating HepG2 cells for 24 h, OA medium (100 μL) at concentrations (100, 200, 300, 400 and 500 μM) and ZFP2 medium (100 μL) at concentrations (200, 400, 600, 800, 1000 μg/mL) were added to the wells, cultured for another 24 h. Cell viability was assessed using the MTT assay kit. Cellular TC was measured using a total cholesterol assay kit. The relationship between the concentrations of OA and ZFP2 and their toxicity was detailed in the supplementary materials.

#### 2.5.3 Oil Red O staining and extraction

Refer to previous studies (Zhang et al., 2017), the blank control group received 2 mL of regular culture medium, while the model group received 2 mL of OA medium (400 μM). The experimental groups received 1 mL of ZFP2-containing culture medium at varying concentrations (600, 800 and 1000 μg/mL) and 1 mL of OA medium. Staining was performed using an Oil Red O staining kit (Beijing Solaibao Technology Co., LTD, Beijing, China) for observation.

#### 2.5.4 Lipid and peroxidation assays

Refer to previous studies (Jiang et al., 2020), after induction with OA treatment, the cells were subsequently treated with ZFP2 (600, 800, and 1000 μg/mL) for 24 h. Cellular contents of TG, TC, LDLC and HDLC, soluble MDA, T-AOC and SOD were determined using commercial kits according to the manufacturer's instructions. The lipid and peroxidation levels were normalized to protein concentration.

#### 2.5.5 RNA extraction and real-time quantitative polymerase chain reaction (RT-qPCR)

Refer to previous studies (Zhang et al., 2017), after incubating HepG2 cells for 24 h, 1 mL of Trizol reagent (Nanjing Nuoweizan Biotechnology Co., LTD, Nanjing, China) was added to fully lyse the cells. The lysate was mixed with 200 μL of precooled chloroform, followed by centrifugation at 13000 ×*g* for 15 min. Collect the upper colorless aqueous phase, where an equal volume of precooled isopropanol was added. The mixture was centrifuged at 13000 ×*g* for 5 min, and the supernatant was discarded. The resulting pellet was air-dried for 2–5 min, then dissolved in 10–20 μL of DEPC-treated water.

Primers were designed using Snapgene 6.0.2 software, with specific details as outlined in the supplementary Table S1. The extracted RNA was used for first-strand cDNA synthesis using a reverse transcription kit. The resulting template DNA was subjected to qRT-PCR using the Hieff qPCR SYBR Green Master Mix (No Rox), following the manufacturer's instructions. Relative gene expression levels of target genes were quantified using the 2^(-ΔΔCT)^ method. β-actin was used as the internal reference gene.

#### 2.5.6 Western blot analysis

Refer to previous studies (Jiao et al., 2020), HepG2 cells were lysed with a lysis buffer prepared by adding 10 μl PMSF (100 mM) to 1 mL RIPA buffer. Lysis was carried out for 30 min on ice, followed by centrifugation at 13000 ×*g* for 5 min at 4 °C. The supernatant was collected and stored at −20 °C. Cells lysates were separated by SDS-PAGE and transferred onto a polyvinylidene fluoride (PVDF) membrane. The membranes were blocked with 5% skim milk in PBS containing 0.1% Tween 20. After blocking, the PVDF membranes were incubated overnight with the specific primary antibodies at 4 °C. The next day, the membranes were washed and incubated with horseradish peroxidase (HRP)-conjugated secondary antibodies. Protein bands were visualized using an ECL substrate (Shanghai Tianneng Life Science Co., LTD, Shanghai, China). Band images were captured using the ChemiDoc™ MP Imaging System (Bio-Rad, Hercules, CA, USA) and the band intensities were analyzed by ImageJ1 software.

### 2.6 Statistical analysis

All experiments were performed in triplicate, and the results were presented as mean ± standard deviation (SD). Data analysis was carried out using one-way analysis of variance (ANOVA) in SPSS 21.0 software, with statistical significance set at *p* < 0.05. Graphs were generated using Origin 2022 and Prism 10 software.

## 3 Results

### 3.1 Isolation and purification of ZFP

**Fig. 1:**
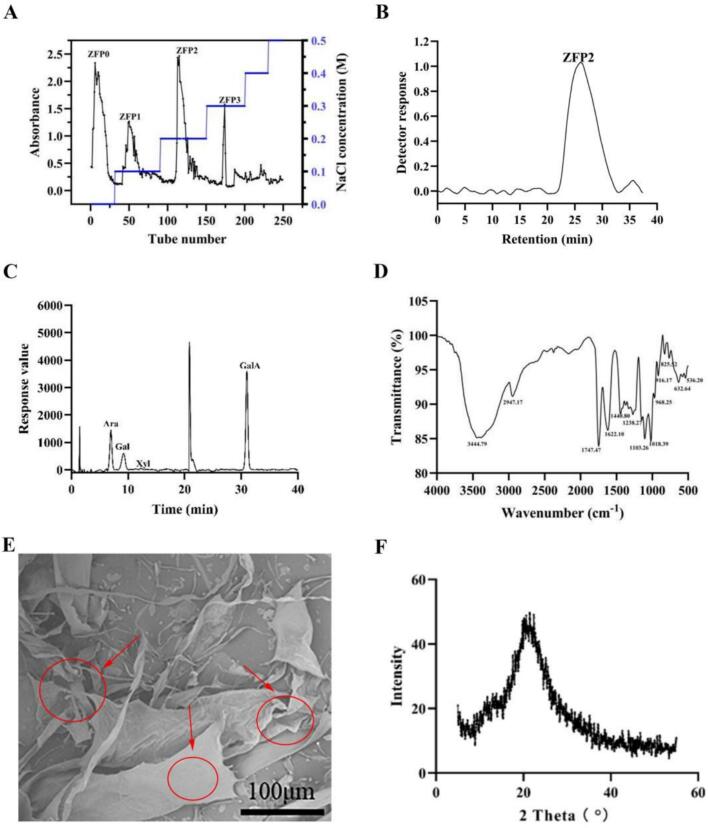
Chromatographies and structural characterization of ZFP2. A: Chromatography of ZFP from fingered citron using a Q sepharose fast flow anion-exchange chromatography column. B: HPGPC of purified ZFP2. C: Ion chromatography of ZFP2 showing its monosaccharide composition. D: FT-IR spectrum of ZFP2, illustrating its functional groups. E: SEM images of ZFP2; F: XRD pattern of ZFP2.

The elution profile of crude polysaccharide ZFP is shown in Fig. 1A. Four distinct elution peaks were observed during the purification process, corresponding to fractions named ZFP0, ZFP1, ZFP2, and ZFP3, with respective yields of 33.79%, 5.16%, 37.21%, and 23.84%. ZFP2 was selected for further purification using Sepharose CL-6B gel column chromatography because it exhibited the highest yield. The elution profile of ZFP2 presented a single symmetrical peak, indicating it was a polysaccharide with uniform molecular weight distribution. The total sugar content of ZFP2 was 44.87% ± 3.46%, the uronic acid content was 48.08% ± 1.20%, and the residual protein content was 0.30% ± 0.22%.

The uronic acid content in ZFP2 is extremely high, which interferes with the determination of total sugar content via the phenol‑sulfuric acid method. Existing literature has analyzed the influence of monosaccharide composition on the quantitative determination of total sugar content via the phenol‑sulfuric acid method and provided correction factors (Yue et al., 2022). Among these, the correction factors for arabinose, galactose, and galacturonic acid are 0.98, 1.01, and 2.13, respectively. This indicates that in the results obtained by the phenol‑sulfuric acid method, the content of neutral sugars such as arabinose and galactose can be determined accurately. In contrast, the measured content of galacturonic acid accounts for only 46.95% of its actual content. Since we have determined via the carbazole‑sulfuric acid method that the content of galacturonic acid is approximately 48.08%, and the result measured by the phenol‑sulfuric acid method is 44.87%, calculations show that the content of neutral sugars (e.g., arabinose and galactose) is 22.30%. Consequently, the actual total sugar content should be 70.38%, which included both uronic acids and neutral sugars.

### 3.2 Characterization of ZFP2

#### 3.2.1 Molecular weight and monosaccharide composition

The molecular weight of ZFP2 is shown in Fig. 1B. The HPGPC analysis of ZFP2 revealed a main absorption peak at 26 min. Using dextran as the standard, molecular weight calibration curves were constructed, and the estimated values of Mw and Mn were 407 kDa and 396.27 kDa, respectively. By comparing retention times and peak areas with standard monosaccharides (Fig. 1C), ZFP2 primarily consisted of arabinose (Ara), galactose (Gal), and galacturonic acid (GalA) in a molar ratio of 2.61:2.27:14.39. No significant amounts of other monosaccharides were detected.

Wang et al. used an ultrasound-assisted enzymatic method to extract fingered citron polysaccharides, precipitating three crude polysaccharide fractions (FCP20, FCP40, FCP60) using varying ethanol concentrations (Wang et al., 2024). These fractions, primarily composed of galacturonic acid and arabinose, were not further purified. However, their results showed that the molecular weight of the polysaccharides decreased with increasing ethanol concentration, being 453.80 kDa, 224.19 kDa, and 215.58 kDa, respectively. The polysaccharide precipitated with 80% ethanol in this study exhibited a molecular weight of 407 kDa. This difference can be mainly attributed to the samples being from different regions as well as the differences in extraction and purification methods. It is speculated that ultrasound and pectinase may cause the cleavage of glycosidic bonds, thereby reducing the molecular weight of fingered citron polysaccharides. He et al. used hot water extraction and purified four fingered citron polysaccharides (FCp-1, FCp-2, FCp-3, and FCp-4) with molecular weights of 113.9, 32.6, 140.3, and 177.1 kDa, respectively (He et al., 2014). Among them, FCp-1 is mainly composed of arabinose, galactose, glucose, rhamnose, and xylose; FCp-2 and FCp-4 only contain galacturonic acid; and FCp-3 contains glucose. It is speculated that this may be due to the boiling treatment of the fruits for 3 h before extraction, which caused the cleavage of some glycosidic bonds, leading to the observed differences. Peng et al. extracted fingered citron polysaccharides by alkali treatment and further purified to obtain CMSPB80-1 with a molecular weight of only 103 kDa (Peng, Yang, et al., 2019). The polysaccharide obtained by alkali extraction in their study, similar to ours, contains arabinose and galactose as the main components but does not contain uronic acid, which is caused by alkali extraction.

#### 3.2.2 Functional group assay by FT-IR spectrum analysis

The FT-IR spectrum of ZFP2 is depicted in Fig. 1D. Spectrum analysis revealed absorption peaks corresponding to various functional groups. The peak at 3444.79 cm^−1^ corresponds to the stretching vibrations of O—H bonds, a predominant functional group in polysaccharides (Xue et al., 2020). The absorption at 2947.17 cm^−1^ indicates methylene stretching vibrations, while the peaks at 1747.47 cm^−1^ and 1622.10 cm^−1^ are attributed to stretching vibrations of carboxylate ions (Yang et al., 2023). The peak at 1103.26 cm^−1^ corresponds to the characteristic absorption of uronic acids in polysaccharides, while the peaks at 1440.80 cm^−1^ and 1238.27 cm^−1^ are associated with -OH bending vibrations (Hongliang et al., 2016). Additionally, the absorption peak at 1018.39 cm^−1^ in the 1200–1000 cm^−1^ region reflects the stretching vibrations of glycosidic bonds (C—O—C) and C—O bending vibrations of hydroxyl groups, suggesting the presence of pyranose rings in ZFP2 (Shuhui et al., 2023). Absorption peaks in the 825–835 cm^−1^ range indicates the presence of α-glycosidic bonds, while a peak at 916.17 cm^−1^ suggests the presence of β-glycosidic bonds (Li et al., 2021) in ZFP2.

#### 3.2.3 Identification of major sugar residues by NMR analysis

**Fig. 2:**
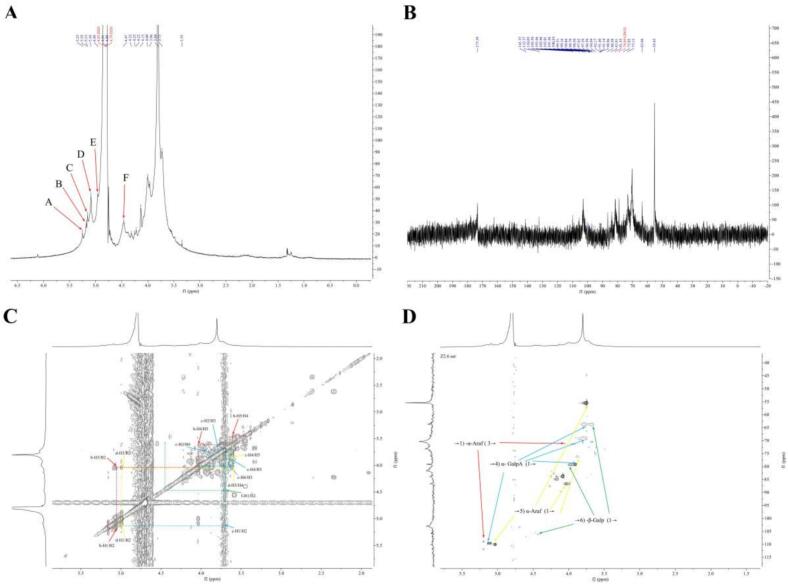
NMR spectra of ZFP2. A: ^1^H NMR spectrum. B: ^13^C NMR spectrum. C: ^1^H—^1^H COSY spectrum. D: HSQC spectrum.

The NMR spectra of ZFP2 (^1^H NMR, ^13^C NMR, COSY, and HSQC) are shown in Fig. 2. The ^1^H signals appeared between δ3.45–6.0 ppm, with α-anomeric protons in the δ5–6 ppm range and β-anomeric protons in the δ4–5 ppm range. A resonance peak at δ4.78 ppm corresponds to D_2_O.

The ^13^C signals were predominantly located between δ60–110 ppm, with anomeric carbons (δ90–110 ppm) and non-anomeric carbons (δ60–85 ppm), characteristic of polysaccharides. In ^1^H NMR, α-glycosidic bonds were identified by anomeric carbon shifts >δ4.90 ppm, while β-glycosidic bonds appeared <δ4.90 ppm. Signals between δ3.45–4.35 ppm were assigned to ring protons (H2–H6), and the absence of signals near δ5.4 ppm confirmed pyranose sugars (Yang et al., 2023).

The ^13^C spectrum showed β-glycosidic bonds with anomeric carbon shifts >100 ppm and α-glycosidic bonds with lower shifts. Combined ^1^H and ^13^C NMR analysis confirmed both α- and β-configurations, with a predominance of α-linkages, consistent with FT-IR results. A characteristic peak at δ173 ppm indicated carboxyl groups, confirming the presence of acidic polysaccharides.

The ^1^H NMR spectrum of ZFP2 (Fig. 2A) revealed six distinct anomeric proton signals at δ5.25, δ5.18, δ5.13, δ5.10, δ4.96, and δ4.47 ppm, designated as A-F, respectively. Signal intensities were correlated with the molar composition of monosaccharides. The ^13^C NMR spectrum (Fig. 2B) displayed broad shifts with weak signals in the anomeric carbon region (δ90–110 ppm).

In the ^1^H—^13^C HSQC spectrum (Fig. 2D), four major anomeric carbon-proton cross-peaks were identified and matched with ^1^H NMR shifts: δ5.18/108.95 ppm (B), δ5.10/109.59 ppm (C), δ5.13/109.55 ppm (D), and δ4.47/105.46 ppm (F). Signals A and E were not resolved in HSQC due to overlap and weak intensity.

Assignments for H2-H6 were deduced from ^1^H—^1^H COSY (Fig. 2C), and corresponding C2-C6 shifts were confirmed via HSQC. Identified residues are summarized in Table 1. Key residues identified were α-L-Araf (Residue B), α-GalAp (Residue C), and β-Galp (Residue F), forming the main structural components of ZFP2 (Farhadi, 2017; Li et al., 2021). Signals at δ5.25 and δ4.96 ppm were attributed to Ara and Glu residues but remain unassigned due to low signal intensity. Additional minor monosaccharides, suggested by the composition analysis, were likely undetected due to low abundance, potentially contributing to side-chain glycosidic linkages.

**Table 1:** ^1^H and ^13^C NMR chemical shifts of major sugar residues in ZFP2.

		chemical shifts(ppm)					
		H1/C1	H2/C2	H3/C3	H4/C4	H5/C5	H6/C6
B	→3) -α-L-Ara*f*- (1→	5.18	5.08	4.02	3.61	3.46	–
		108.95	63.69	69.17	69.21	–	–
C	→4) -α-D-Gal*p*A- (1→	5.13	3.70	3.75	3.86	3.64	4.00
		109.55	63.69	69.17	69.21	63.69	79.13
D	→5) -α-L-Ara*f*- (1→	5.10	4.99	4.03	3.61	3.87	–
		109.59	–	86.62	72.53	76.23	–
F	→6) -β-D-Gal*p*- (1→	4.47	3.68	4.00	3.61	4.02	3.87
		105.46	63.76	79.17	72.53	86.64	76.23

#### 3.2.4 Identification of bond types of sugar residues by methylation and GC–MS analysis

Methylation analysis is an effective method for identifying the type of bond between sugar residues. The total ions chromatogram of ZFP2 was shown in Fig. S5. According to the relative retention time and mass spectrometry of each peak, the literature and the ion fragment mass spectrometry of the Complex Carbohydrate Research Center database of the University of Georgia, the type of saccharide residue corresponding to each peak were analyzed, and the peak area of the corresponding sugar residue type was used to calculate its content. The results are shown in Table 2. The results revealed that the predominant linkage type in ZFP2 was 1,4-GalpA (43.727%), most likely derived from the high galacturonic acid (HG) domain. Additionally, ZFP2 have RG-I domains, whose backbone consists of 1,2,4-Rhap (3.038%), 1,5-Araf (12.422%), 1,3,5-Araf (1.368%), 1,3-Galp (2.806%), 1,6-Galp (4.122%), 1,3,6-Galp (9.271%), and 1,3,4-GalpA (0.897%) indicated the presence of arabinogalactan I and II (AG-I and AG-II) branches of RG-I (Qiao et al., 2024). While methylation and GC–MS analysis effectively characterizes glycosidic linkage patterns, its quantitative limitations necessitate integration of monosaccharide and NMR profiling for precise structural elucidation.

$$\text{Relative molar weight}=\frac{\text{Peak area}}{\text{Molecular weight}}$$

$$\text{Relative molar ratio}\;\left(\%\right)=\frac{\text{Relative molar weight}}{\text{The}\;\mathrm{sum}\;\text{of relative molar weight of each component}}$$

**Table 2:** GC–MS data for methylation analysis of ZFP2.

RT(min)	Polymethacrylic acid derivatives	Relative molar ratio (%)	Glycosidic linkage
11.726	1,4-di-*O*-acetyl-2,3,5-tri-*O*-methyl arabinitol	6.711	t-Araf
12.758	1,5-di-*O*-acetyl-6-deoxy-2,3,4-tri-*O*-methyl rhamnitol	1.854	t-Rhap
15.473	1,4,5-tri-*O*-acetyl-2,3-di-*O*-methyl arabinitol	12.422	1,5-Araf
15.655	1,4,5-tri-*O*-acetyl-2,3-di-*O*-methyl xylitol	0.863	1,4-Xylp
16.781	1,5-di-*O*-acetyl-2,3,4,6-tetra-*O*-methyl glucitol	1.976	t-GlcpA
17.487	1,5-di-*O*-acetyl-2,3,4,6-tetra-*O*-methyl galactitol	9.492	t-GalpA
17.982	1,3,4,5-tetra-*O*-acetyl-2-*O*-methyl arabinitol	1.368	1,3,5-Araf
18.401	1,2,4,5-tetra-*O*-acetyl-6-deoxy-3-*O*-methyl rhamnitol	3.038	1,2,4-Rhap
19.852	1,4,5-tri-*O*-acetyl-2,3,6-tri-*O*-methyl galactitol	43.727	1,4-GalpA
20.346	1,3,5-tri-*O*-acetyl-2,4,6-tri-*O*-methyl galactitol	2.806	1,3-Galp
21.578	1,5,6-tri-*O*-acetyl-2,3,4-tri-*O*-methyl galactitol	4.122	1,6-Galp
21.991	1,3,4,5-tetra-*O*-acetyl-2,6-di-*O*-methyl galactitol	0.897	1,3,4-GalpA
22.579	1,2,4,5-tetra-*O*-acetyl-3,6-di-*O*-methyl galactitol	0.885	1,2,4-GalpA
23.555	1,4,5,6-tetra-*O*-acetyl-2,3-di-*O*-methyl galactitol	0.567	1,4,6-GalpA
24.418	1,3,5,6-tetra-*O*-acetyl-2,4-di-*O*-methyl galactitol	9.271	1,3,6-Galp

#### 3.2.5 Microstructural characterization by SEM analysis

The microstructure of ZFP2 is depicted in Fig. 1E. ZFP2 exhibited irregular flake-like structures, with some areas interwoven with filamentous structures. Such features may increase the surface area of the polysaccharide, thereby enhancing its solubility.

#### 3.2.6 Crystalline structure characterization by XRD analysis

The XRD pattern of ZFP2 is shown in Fig. 1F. A broad diffraction peak was observed around 20° within the range of 5–55°, representing the primary crystalline reflection region of ZFP2. The relative crystallinity of the polysaccharide was calculated using the Segal method (Cheng et al., 2024), with the calculated value being 32.21%. However, the low intensity of the diffraction peaks suggests weak crystallinity. Consequently, ZFP2 existed predominantly in a semi-crystalline form with a low crystalline proportion. This property likely contributes to its enhanced solubility and bioavailability.

### 3.3 Hypolipidemic activity of ZFP2

#### 3.3.1 Inhibitory effect of ZFP2 on lipid profiles of oleic acid-induced high-fat HepG2 cells

**Fig. 3:**
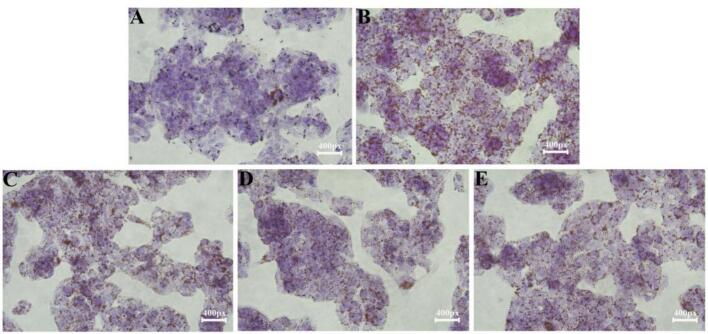
Effects of ZFP2 treatment on OA-induced lipid deposition in HepG2 cells (Oil Red O Stain, A: Control, B: OA, C-E: ZFP2 of 600, 800 and 1000 μg/mL). (For interpretation of the references to color in this figure legend, the reader is referred to the web version of this article.)

The effect of ZFP2 on lipid droplet accumulation in HepG2 cells is shown in Fig. 3A-E. The control group exhibited no noticeable lipid droplets, while cells treated with OA showed abundant lipid droplets, significantly increased in number and stained the cells. In contrast, the ZFP2-treated group exhibited markedly reduced lipid staining, with significantly fewer lipid droplets that were lighter in color and smaller stained areas. These results indicates that ZFP2 significantly reduced lipid accumulation and inhibited lipid fusion in HepG2 cells.

#### 3.3.2 Effect of ZFP2 on lipid profiles of oleic acid-induced high-fat HepG2 cells

**Fig. 4:**
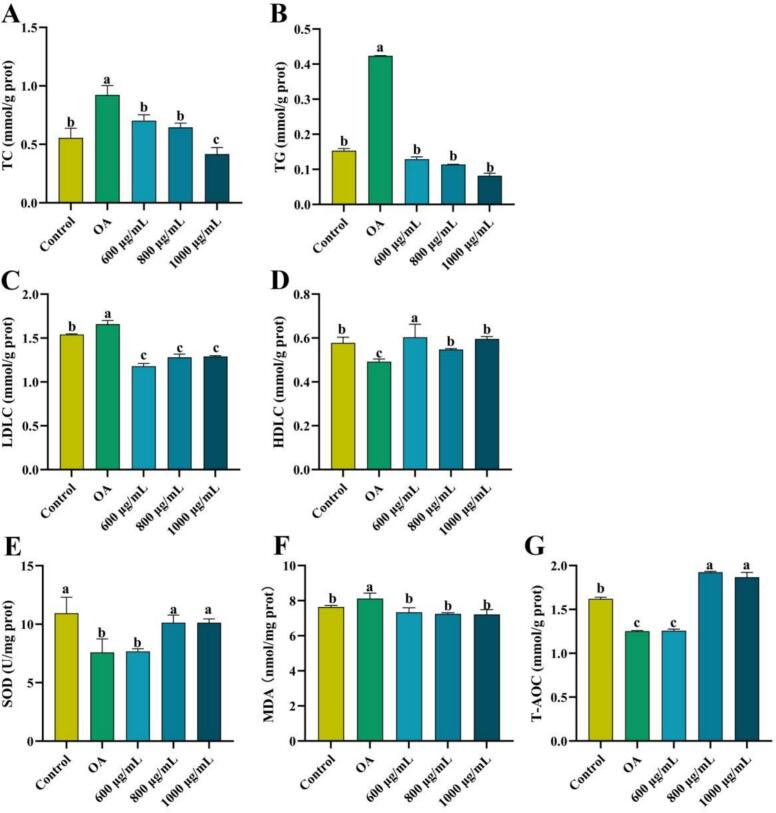
ZFP2 ameliorates lipid spectrum and oxidative stress in OA-induced steatosis cells. A-D: Effects of ZFP2 on TC (A), TG (B), LDLC (C), HDLC (D) levels in HepG2 cells. *E*-G: Effects of ZFP2 on SOD (E), MDA (F), T-AOC (G) in HepG2 cells. Error bars are mean ± SD. Different lowercase letters indicate significant differences between groups (*p* < 0.05).

Fig. 4A-D illustrates the influence of different treatments on lipid profile of the OA-induced high-fat HepG2 cells. Compared to the control group, the model group showed significant increases in TC, TG, and LDLC levels, along with a significant decrease in HDLC levels (*p* < 0.05), indicating impaired lipid metabolism post OA treatment. However, treatment with different doses of ZFP2 reduced intracellular lipid accumulation in a dose-dependent manner. At 1000 μg/mL, ZFP2 reduced TC and TG levels by 53.42% and 80.52%, respectively. At 600 μg/mL, ZFP2 decreased LDLC levels by 28.49% and increased HDLC levels by 18.82%. These results demonstrate that ZFP2 can significantly improve the lipid profile of high-fat HepG2 cells.

#### 3.3.3 Effect of ZFP2 on oxidative stress of oleic acid-induced high-fat HepG2 cells

As shown in Fig. 4E-G, the OA group exhibited significantly decreased SOD and T-AOC levels, with a significant increase in MDA content (*p* < 0.05) compared to the control group, indicating oxidative damage. In contrast, the ZFP2-treated groups decreases in MDA content, along with significant increases in SOD activity and T-AOC (*p* < 0.05). In contrast, the MDA content decreased by 11.14%, and the SOD and T-AOC levels increased by 33.41% and 49.11% at 1000 μg/mL, respectively, when compared to the OA group. Hyperlipidemia often induces oxidative stress, leading to cellular damage. ZFP2 can enhance the activity of endogenous antioxidant enzymes and improve overall antioxidant capacity, thereby reducing the production of oxidative products and playing a role in alleviating oxidative stress.

#### 3.3.4 Effect of ZFP2 on genes involved in lipid metabolism of oleic acid-induced high-fat HepG2 cells

**Fig. 5:**
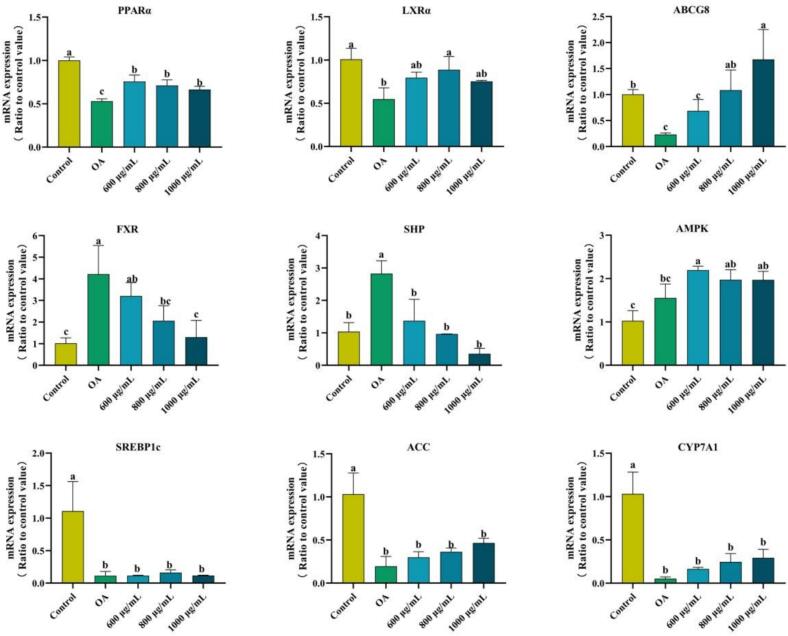
Effects of ZFP2 on relative gene expression contents in HepG2 cells. Error bars are mean ± SD. Different lowercase letters indicate significant differences between groups (*p* < 0.05).

The effect of ZFP2 on the mRNA expression levels of key lipid metabolism factors is depicted in Fig. 5. Compared to the control group, OA treatment significantly decreased the expression of *PPARα*, *LXRα*, and *ABCG8*, while increasing the mRNA levels of *SHP* and *FXR* (*p* < 0.05). There was no significant impact observed in the expression of on *AMPK* expression levels, but *ACC* and *CYP7A1* factors showed a reverse inhibitory effect (*p* < 0.05). Treatment with ZFP2 alleviated OA-induced changes by increasing the mRNA expression of *PPARα*, *LXRα*, and *ABCG8* by 34.11%, 61.99% and 368.73% at 800 μg/mL, respectively, and decreasing the levels of *FXR* and *SHP* (*p* < 0.05) by 69.26% and 87.46% at 1000 μg/mL, respectively, compared to the OA group.

**Fig. 6:**
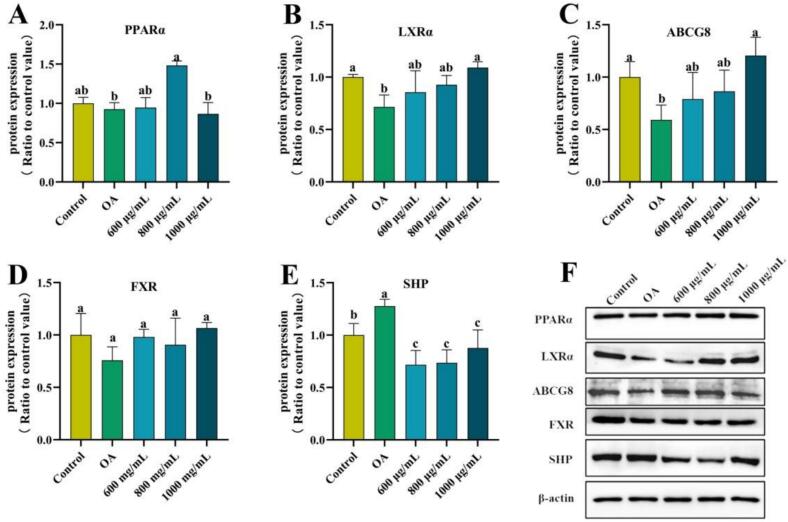
Effects of ZFP2 on relative protein expression contents in HepG2 cells. Error bars are mean ± SD. Different lowercase letters indicate significant differences between groups (*p* < 0.05).

Western blot analysis was further conducted to assess the protein levels of PPARα, LXRα, ABCG8, FXR, and SHP, as depicted in Fig. 6. Compared to the control group, the OA group showed decreased protein levels of LXRα and ABCG8, along with increased SHP protein level (*p* < 0.05). In contrast, the ZFP2-treated group exhibited enhanced protein expression of PPARα (800 μg/mL), LXRα (1000 μg/mL), and ABCG8 (1000 μg/mL) by 60.19%, 52.38%, 103.73%, respectively, while SHP protein levels (600 μg/mL) were significantly reduced by 43.91% (*p* < 0.05). FXR protein level remained unchanged across all groups.

ZFP2 upregulated the expression of *PPARα*, *LXRα*, and *ABCG8*, suggesting that its lipid-lowering mechanism involved the *PPARα/LXRα/ABCG8* signaling pathway. *PPARα* regulates fatty acid metabolism and cholesterol homeostasis through *LXRα*, which controls *ABCG8*-mediated cholesterol efflux. The activation of this pathway may promote HDLC synthesis, cholesterol catabolism, and reverse cholesterol transport, reducing cholesterol accumulation.

## 4 Discussion

**Fig. 7:**
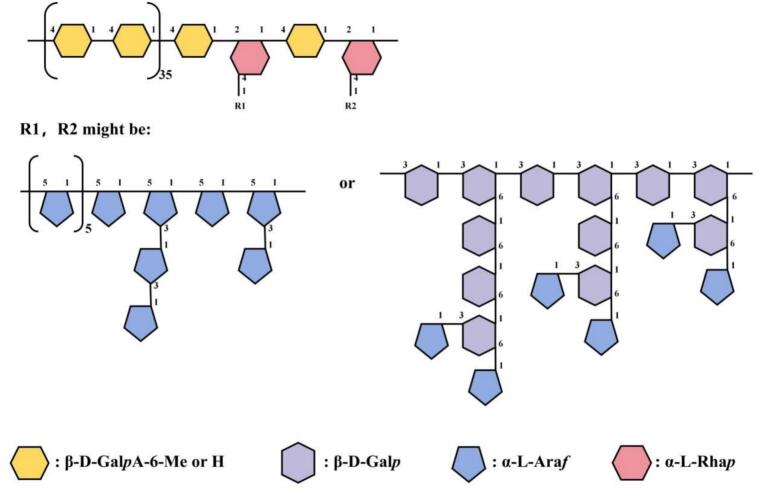
The polysaccharide structure composition of ZFP2 (Polygons of different shapes and colors represented different types of monosaccharides; numbers indicated the linkage types of glycosidic bonds).

The polysaccharide ZFP2 isolated from fingered citron in this study was mainly composed of Ara, Gal, and GalA with a molar ratio of 2.61:2.27:14.39, indicating that the backbone of ZFP2 was dominated by GalA. Structural analysis of ZFP2 revealed that its sugar ring configurations include both α- and β-pyranoses. NMR analysis revealed that the main sugar residue fragments of ZFP2 include [→1)-α-Araf(3→], [→4)α-GalAp(1→], [→5)α-Araf(1→], and [→6)-β-Galp(1→]. Methylation analysis revealed that the predominant linkage type in ZFP2 was 1,4-GalpA (43.727%). Its backbone consists of 1,2,4-Rhap (3.038%), 1,5-Araf (12.422%), 1,3,5-Araf (1.368%), 1,3-Galp (2.806%), 1,6-Galp (4.122%), 1,3,6-Galp (9.271%), and 1,3,4-GalpA (0.897%) indicated the presence of arabinogalactan I and II (AG-I and AG-II) branches. Studies have shown that in citrus polysaccharides (Liang et al., 2025), GalA typically forms the backbone via α-(1 → 4) glycosidic linkages, and the side chains may further contain complex branched structures composed of Ara and Gal. Additionally, [→6)-β-Galp(1→] and [→3)-β-Galp(1→] are often detected in arabinogalactans (Yao et al., 2021). This suggests that ZFP2 may form its backbone through [→4)α-GalAp(1→], with arabinogalactan branches formed via [→1)-α-Araf(3→], [→5)α-Araf(1→], and [→6)-β-Galp(1→]. Referring to previous studies (Pérez et al., 2000) it can be inferred that the backbone of ZFP2 is mainly a homopolymer of (1 → 4)-α-D-galacturonic acid, and its side chains mainly consist of arabinan and galactan. The specific structure of ZFP2 is shown in the Fig. 7.

The weight-average molecular weight of ZFP2 was 407 kDa. Previous study showed that high-molecular-weight fractions of polysaccharides from the Citrus genus (CGPs) predominantly distributed around 528.8 kDa, exhibit strong prebiotic activity and intestinal regulatory functions (Liang et al., 2025). These CGPs maintain sufficient molecular chain length while retaining high solubility, making them ideal candidates for pharmaceutical and functional food development.

Ara and Gal were common neutral monosaccharides in CGPs, typically existing in branched structures as side chains or branches from the main chain. Their contents directly affect the solubility, degree of branching, and biological activity of the polysaccharides. Pectin derivatives from CGPs with high Ara and Gal contents display potent immunomodulatory and anti-inflammatory activities. Additionally, the levels of Ara and Gal may regulate the antioxidant capacity of CGPs. GalA, as the main component of CGPs, plays a core role in the backbone structure. Its content and distribution significantly influence the gel-forming properties and biological activities of CGPs (Liang et al., 2025).

Structural features, such as monosaccharide composition, molecular weight, and glycosidic linkage types, influence the biological activity of polysaccharides. For example, lipid-lowering effects have been observed in polysaccharides containing [→3)-α-Araf-(1→] (Liu et al., 2023), [→5)-α-Araf-(1→] (Jiao et al., 2020), and [→4)-β-D-GalpA-(1→] (Li et al., 2023). NMR analysis and Methylation analysis of ZFP2 identified similar sugar residues, including [→1)-α-Araf-(3→], [→4)-α-GalAp-(1→], [→5)-α-Araf-(1→], and [→6)-β-Galp-(1→], resembling pectin-like homotypic galacturonic acid polysaccharides. These structures are known for antioxidative and anti-inflammatory properties (Jin et al., 2021). Hyperlipidemia is closely associated with oxidative stress and inflammatory responses, which indicates that ZFP2 has the potential to alleviate hyperlipidemia.

The XRD results indicate that ZFP2 mainly exists in the form of semi-crystals with a low crystallization rate. The relative crystallinity calculated is 32.21%. Cheng et al. extracted a polysaccharide PCP-80% from Polygonatum sibiricum (Cheng et al., 2024). Their XRD results showed that PCP-80% displayed a relatively pronounced broad crystalline diffraction peak at 2 θ = 13.5°, with a calculated relative crystallinity of 26.87%. This indicated that PCP-80% contains both crystalline and amorphous components, which is similar to our XRD analysis results. Jiang et al. obtained three polysaccharides from sugarcane leaves (Jiang et al., 2023). Their XRD results revealed that in the range of 5°–80°, the diffraction peaks of ASLPs are similar, with a broad diffraction peak formed around 2 θ = 22°. The overall crystallinity is low, and there are no obvious sharp peaks. They suggested that the stepwise alcohol precipitation method may affect the structure of ASLPs. Our XRD results are completely consistent with theirs. Therefore, the stepwise alcohol precipitation method will also affect the structure of ZFPs.

Characteristics in Fig. 1E show that the irregular flake structure and filamentous interwoven morphology of ZFP2 can increase the surface area. This structural feature provides more sites for its contact with solvents, which is theoretically beneficial to improving solubility. The results in Fig. 1F indicate that ZFP2 is mainly in a semi-crystalline form with a low proportion of crystals and weak crystallinity. Low crystallinity means poor orderliness of molecular arrangement and weak intermolecular forces, making it easier to be penetrated and dispersed by solvent molecules, which also helps to enhance solubility. Combining the two, a common conclusion can be drawn: ZFP2 has high solubility, which is conducive to improving its bioavailability. This lays a foundation for subsequent experiments related to biological activity.

The HepG2 hepatocellular carcinoma cell line is widely used in studies on liver function, metabolism, and drug toxicity. HepG2 cells possess biochemical and morphological characteristics of normal hepatocytes, retaining a sufficient number of properties of normal liver cells. The potential advantages of hepatoma cells lie in the fact that, as an immortal cell line, they can be obtained on a large scale, are easy to preserve, and their drug-metabolizing enzyme activity does not decrease during culture as it does in human hepatocytes (González et al., 2017). However, a significant defect of liver cancer cells is that there are deviations in the mechanisms related to drug metabolism and toxicity in the transformed cells, which in turn affects the evaluation of lipid metabolism mechanisms. Subsequent experiments can be conducted on different liver cell lines to make up for this deficiency.

OA, a common dietary fatty acid, can induce lipid accumulation and cellular damage at certain concentrations. Numerous studies have demonstrated that OA can successfully induce high-fat cell models (Xiao et al., 2024). Oil Red O staining confirmed that ZFP2 lipid droplet formation in liver cells, though no significant differences were observed among concentrations (Jiao et al., 2020). ZFP2 reduced TC, TG, and LDLC, while increasing HDLC levels, indicating its lipid-lowering effects. Additionally, ZFP2 improved oxidative stress by increasing SOD and T-AOC levels and decreasing MDA levels, suggesting its ability to mitigate OA-induced lipid accumulation and associated oxidative stress. Quantitative RT-PCR and Western blot analyses revealed that ZFP2 upregulated the expression of *PPARα*, *LXRα*, and *ABCG8*, suggesting that its lipid-lowering mechanism involved the *PPARα/LXRα/ABCG8* signaling pathway. *PPARα* regulates fatty acid metabolism and cholesterol homeostasis through *LXRα*, which controls *ABCG8*-mediated cholesterol efflux (Xu et al., 2017). The activation of this pathway may promote HDLC synthesis, cholesterol catabolism, and reverse cholesterol transport, reducing cholesterol accumulation. Interestingly, ZFP2 did not significantly affect the expression of key lipid synthesis genes (*acc*, *fas*, and *srebp1c*), indicating its lipid-lowering effects were primarily through lipid degradation rather than synthesis. ZFP2 also inhibited the *FXR/SHP* pathway, potentially enhancing bile acid synthesis and cholesterol consumption. In summary, ZFP2 can alleviate hyperlipidemia by improving oxidative stress and promoting lipid catabolism through the *PPARα/LXRα/ABCG8* signaling pathway. This study only analyzed the mechanisms from the perspectives of oxidative stress and lipid metabolism. However, polysaccharides are highly likely to exert their effects by regulating the composition of the gut microbiota and its metabolites. Therefore, further research is still needed to fully clarify the mechanism by which ZFP2 alleviates hyperlipidemia.

The active concentration of ZFP2 in this study (600–1000 μg/mL) can directly act on HepG2 cells in vitro, but extrapolation to humans requires caution. Given the low absorption rate of polysaccharides, this concentration may only reflect local intestinal effects or indirect actions of metabolites. Based on the extraction yield of ZFP2 from fingered citron, a daily intake of 0.6–1 g of purified ZFP2 (equivalent to 30–50 g of dried raw material) could achieve an equivalent dose. However, the actual efficacy may depend on transformation by intestinal flora or regulation via the gut-liver axis, and further animal experiments are needed to verify the effectiveness of this dosage.

## 5 Conclusion

In conclusion, a novel polysaccharide, ZFP2, was successfully isolated from fingered citron. Monosaccharide composition analysis showed that ZFP2 consisted of Ara, Gal, and GalA with molar ratios of 2.61:2.27:14.39, with a weight-average molecular weight of 407 kDa. The majorly backbone of ZFP2 is composed of 1,4-GalpA, 1,2,4-Rhap, 1,5-Araf, 1,6-Galp and 1,3,6-Galp. Structural analysis indicated that ZFP2 existed in a semi-crystalline state with an irregular plate-like morphology. ZFP2 significantly reduced lipid accumulation and oxidative stress in OA-treated HepG2 cells, regulating the expression of key genes and proteins involved in lipid metabolism. Its effects were particularly evident in the *PPARα/LXRα/ABCG8* and *SHP*-related pathways, highlighting its potential in improving hyperlipidemia. These findings support the development of functional foods incorporating fingered citron polysaccharides and promote the comprehensive utilization of citrus fruits for health-related applications. Further research on fingered citron polysaccharides will provide deeper insights into the bioactivity and therapeutic potential of homologous resources for medicine and food.

## CRediT authorship contribution statement

**Chong Wu:** Writing – review & editing, Writing – original draft, Visualization, Validation, Supervision, Formal analysis, Data curation. **Lianger Dong:** Writing – review & editing, Writing – original draft, Supervision, Project administration. **Jinhua Zhou:** Writing – original draft, Methodology, Investigation, Formal analysis, Data curation. **Sibao Wan:** Supervision, Resources, Project administration. **Zhen Qin:** Supervision, Resources, Project administration. **Haiyan Gao:** Supervision, Resources, Project administration, Funding acquisition, Conceptualization.

## Declaration of competing interest

The authors declare that they have no known competing financial interests or personal relationships that could have appeared to influence the work reported in this paper.

## Data Availability

Data will be made available on request.

## Appendix A Supplementary data

Supplementary material Image 1
