# Supplementary material for: A novel polysaccharide of Citrus medica L. var. sarcodactylis Swingle: purification, structural characterization, and hypolipidemic effects
Source: Food Chem X. 2026 Jan 17;33:103559. doi: 10.1016/j.fochx.2026.103559 (PMC12853050; doi:10.1016/j.fochx.2026.103559)
Supplement: Supplementary file 1 — Supplementary material [file mmc1.docx]

**Supplementary Information**

**A novel polysaccharide of** ***Citrus medica* L. var. *sarcodactylis* Swingle: Purification, structural characterization, and hypolipidemic effects**

Chong Wu, Lianger Dong, Jinhua Zhou, Sibao Wan, Zhen Qin*, Haiyan Gao*

School of Life Sciences, Shanghai University, Shanghai, 200444, China

*Email: hygao1111@126.com

*Email: qin_zhen@shu.edu.cn

**Supplementary table S1**

Table S1 Primers of key genes involved in lipid metabolism.

| Gene name | Primer sequence (5 '-3') | |
| --- | --- | --- |
| β-actin | Forward | ATGGGTCAGAAGGATTCCTATGT |
|  | Reverse | GTAGAAGGTGTGGTGCCAGATTT |
| AMPK | Forward | GCTCCCCTGCCAAATTATTCAA |
|  | Reverse | TTCCT*ACC*ACATCAAGGCTCCG |
| ACC | Forward | AGAGGATGAGATCAGCA*ACC*TG |
|  | Reverse | TTTATTTCCCCCAAAGCGAGTA |
| FAS | Forward | GCCAATTCTGCCATAAGCCC |
|  | Reverse | ACGCAGTCTGGTTCATCCCC |
| LXRα | Forward | *ACC*ATCCTCTTCTCCCAGCAAG |
|  | Reverse | T*ACC*AAGGCACTGTCCAAATCC |
| FXH | Forward | GAAAGAAAAAGGAAGGAAAACGG |
|  | Reverse | CAC*ACC*TCCACAGCTTTCTTTTC |
| SHP | Forward | GAGCTGGAAGTGAGAGCAGATCC |
|  | Reverse | TCAGAAGTGCGTAGAGAATGGCG |
| ABCG8 | Forward | GCTGGGTCTAAGAGAGCTGCAG |
|  | Reverse | GATGTCCAGGGCATCTTGAACT |
| CYP7A1 | Forward | TGGCATCCTTCCCTTTCTAATCA |
|  | Reverse | TC*ACC*CGTTTGCCTTCTCCTAAT |
| SREBP1 | Forward | CCATGGATTGCACTTTCGAAGA |
|  | Reverse | CCAGCATAGGGTGGGTCAAATA |
| PPARα | Forward | GCGATGGTGGACACGGAAA |
|  | Reverse | AGGAGGGGCTCGAAGCTGG |

Note: Adenosine 5 '-monophosphate (*AMP*)-activated (*AMPK*), ATP binding cassette subfamily G member 8 (*ABCG8*), Small heterodimer partner (*SHP*).

**Supplementary Figure S1**


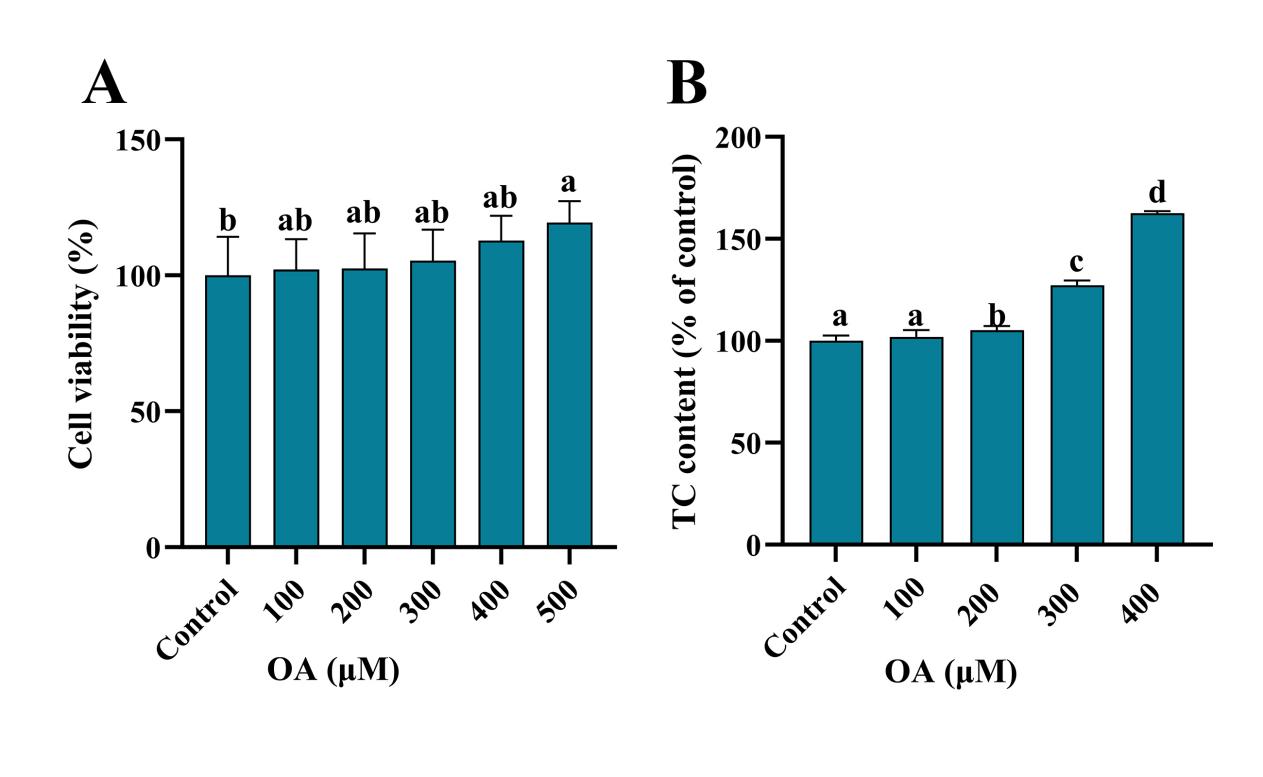


Fig. S1 Effect of OA on HepG2 cell viability and lipid accumulation A: Effects of OA on cell viability; B: Effects of OA on TC content in HepG2 cells; Different lowercase letters indicate significant differences between groups (p < 0.05).

The effects of different concentrations of OA on HepG2 cell viability are shown in Fig. S1A. In the concentration range of 100-400 μM, no significant difference in cell viability was observed compared to the control group, with cell viability remaining above 95% (p < 0.05). However, at 500 μM, OA significantly enhanced cell proliferation (p < 0.05). Therefore, OA concentrations of 100-400 μM, which had minimal impact on cell viability, were selected for subsequent experiments. The effect of OA (100-400 μM) on cellular TC content is shown in Fig. S1B. Compared to the control group, TC content increased with the increasing of OA concentration, indicating that OA effectively induced lipid accumulation in HepG2 cells. Subsequently, 400 μM OA, which induced the most lipid accumulation, was chosen as the modeling concentration.

**Supplementary Figure S2**


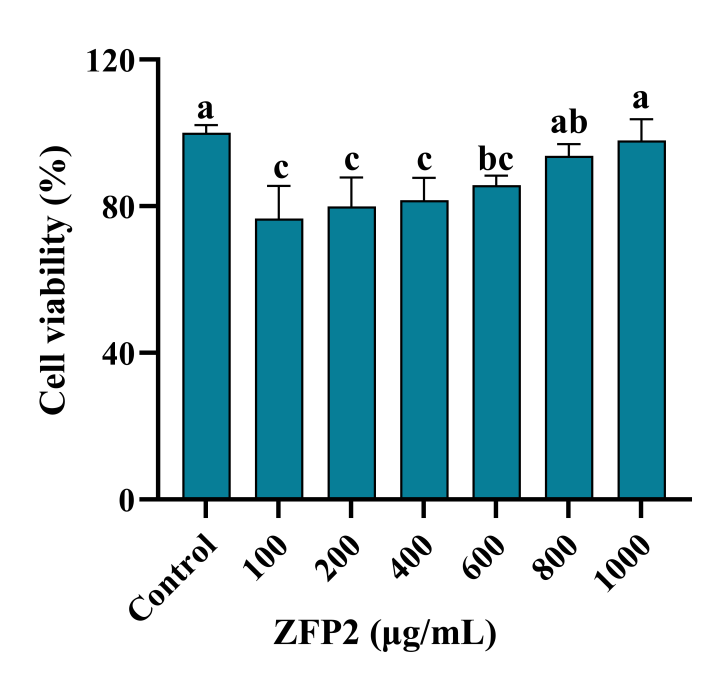


Fig. S2 Effects of ZFP2 on cell viability. Error bars are mean ± SD. Different lowercase letters indicate significant differences between groups (p < 0.05).

The effect of ZFP2 treatment on HepG2 cell viability is depicted in Fig. S2. ZFP2 exhibited varying degrees of inhibitory effects on HepG2 cells within the range of 100 to 1000 μg/mL. Compared to the control group, ZFP2 at concentrations of 600, 800, and 1000 μg/mL showed the least inhibition of cell viability. Therefore, these three concentrations were selected as low, medium, and high doses for subsequent experiments.

**Supplementary Figure S3**

**
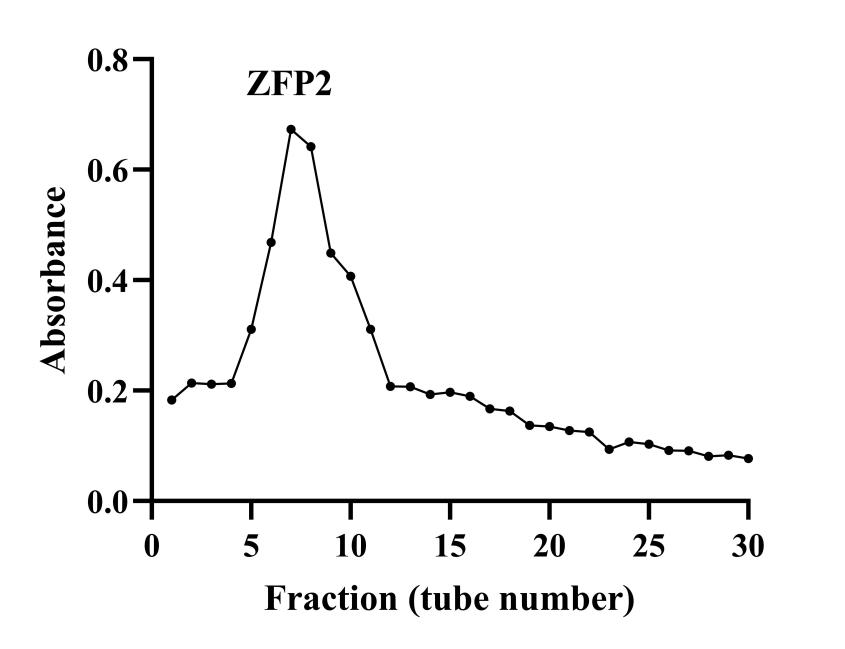
**

Fig. S3 Elution curve of ZFP2 on Q Sepharose CL-6B gel column

**Q Sepharose FF Ion Exchange Chromatography**

- Column Packing:

Secure a cleaned chromatography column (2.6 cm × 40 cm, Shanghai Lianta Instrument Co., Ltd.) onto a chromatography rack. Add a specific volume of ultrapure water to the column, then open the outlet at the bottom to allow slow water flow. Gently pour pre-swollen Q Sepharose FF resin into the column from the top along the inner wall. Allow the resin to settle naturally until the column is filled to two-thirds of its height and the bed volume stabilizes.

- Column Equilibration and Sample Loading:

Equilibrate the column with 5–10 column volumes of ultrapure water at a flow rate of 1 mL/min. Connect the top of the column to an eluent reservoir and the bottom to an automatic fraction collector. After equilibration, load 250 mg of the sample onto the column at 1 mL/min, then let it adsorb for 30 min.

- Elution:

Elute the column sequentially with ultrapure water, 0.1 M, 0.2 M, and 0.3 M NaCl solutions at a flow rate of 2 mL/min.

- Fraction Collection and Processing:

Collect eluent fractions (5 mL/tube) using an automatic fraction collector. Analyze each fraction via the phenol-sulfuric acid method to identify carbohydrate-containing peaks. Pool fractions corresponding to each peak, dialyze, lyophilize, and store at -20 ℃.

**Sepharose CL-6B Gel Filtration Chromatography**

- Column Packing:

Add a small amount of ultrapure water to the bottom of a chromatography column (1.6 cm × 50 cm, Shanghai Lianta Instrument Co., Ltd.). Slowly pour pre-swollen Sepharose CL-6B resin into the column from the top along the inner wall. Allow the resin to settle until the bed volume stabilizes.

- Column Equilibration and Sample Loading:

Equilibrate the column with 5–10 column volumes of ultrapure water at 0.8 mL/min. Load 25 mg of the purified fraction from Q Sepharose FF chromatography onto the column at 0.6 mL/min, then let it adsorb for 30 min.

- Elution:

Elute the column with ultrapure water at a flow rate of 0.6 mL/min.

- Fraction Collection and Processing:

Collect eluent fractions (5 mL/tube) and analyze them using the phenol-sulfuric acid method to detect carbohydrate peaks. Pool relevant fractions, lyophilize, and store at -20 ℃.

**Supplementary Figure S4**

Fig. S4 HPGPC of purified ZFP2

Table S2 Molecular Weight Data of ZFP2

| RT(min) | lgMp | lgMw | lgMn | Mp | Mw | Mn |
| --- | --- | --- | --- | --- | --- | --- |
| 26.01 | 5.6 | 5.6 | 5.6 | 413024 | 407001 | 396269 |

**Molecular Weight of ZFP2**

(1) Standard Solution Preparation

Accurately weigh 2 mg of each of the 8 dextran standards with different molecular weights. Dissolve each standard in 1 mL of the mobile phase solution to prepare a 2 mg/mL solution. Transfer the prepared solution into a 1.8 mL injection vial.

(2) Sample Solution Preparation

Accurately weigh 2 mg of the sample, dissolve it in 1 mL of the mobile phase solution to prepare a 2 mg/mL solution. Sonicate the solution for 10 min, then centrifuge at 13,000 × g for 10 min. Aspirate the supernatant, filter it through a 0.22 μm aqueous microporous membrane, and transfer the filtered solution into a 1.8 mL injection vial.

(3) Selection of Chromatographic Method

Mobile phase: 0.2 M NaCOOH solution

Chromatographic column: Waters Ultrahydrogel Columns 2000-500-250 in series (7.8 × 300 mm); Flow rate: 0.8 mL/min; Column temperature: 40 ºC; Injection volume: 25 μL; Detector: Refractive Index Detector (RID-10A); Analysis time: 60 min

(4) Analysis Procedures

Place the standard solutions on the autosampler tray. Perform analysis using the above-described chromatographic method to obtain retention times (RT). Construct calibration curves for lgMp-RT (peak molecular weight vs. retention time), lgMw-RT (weight-average molecular weight vs. retention time), and lgMn-RT (number-average molecular weight vs. retention time), and derive the corresponding molecular weight calculation formulas.

Place the sample solution on the autosampler tray. Conduct analysis using the same chromatographic method to obtain the chromatogram and retention time. Substitute the sample’s retention time into the derived formulas to calculate the molecular weights (Mp, Mw, Mn).

(5)Experimental Results

The calibration curves for lgMp-RT, lgMw-RT, and lgMn-RT were obtained, with the following equations:

lgMp-RT calibration curve equation: y = -0.2025x + 10.883 (R² = 0.9998)

lgMw-RT calibration curve equation: y = -0.2004x + 10.822 (R² = 0.9997)

lgMn-RT calibration curve equation: y = -0.201x + 10.826 (R² = 0.9996)

Based on the standard calibration curves, the molecular weight calculation formulas were derived, and the molecular weight of the sample was calculated accordingly. The molecular weight chromatogram of the sample is shown in the figure, and the calculation results are summarized in the table.

**Supplementary Figure S5**


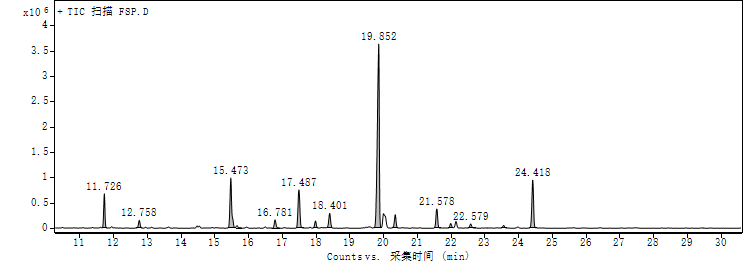


The total ion current diagram of ZFP2
